# p3d – Python module for structural bioinformatics

**DOI:** 10.1186/1471-2105-10-258

**Published:** 2009-08-21

**Authors:** Christian Fufezan, Michael Specht

**Affiliations:** 1 Westfälische Wilhelms-Universität Münster, Institute for evolution and biodiversity, Hüfferstr. 1, 48149 Münster, Germany; 2 Westfälische Wilhelms-Universität Münster, Institut für Biochemie und Biotechnologie der Pflanzen, Hindenburgplatz 55, 48143 Münster, Germany

## Abstract

**Background:**

High-throughput bioinformatic analysis tools are needed to mine the large amount of structural data via knowledge based approaches. The development of such tools requires a robust interface to access the structural data in an easy way. For this the Python scripting language is the optimal choice since its philosophy is to write an understandable source code.

**Results:**

p3d is an object oriented Python module that adds a simple yet powerful interface to the Python interpreter to process and analyse three dimensional protein structure files (PDB files). p3d's strength arises from the combination of a) very fast spatial access to the structural data due to the implementation of a binary space partitioning (BSP) tree, b) set theory and c) functions that allow to combine a and b and that use human readable language in the search queries rather than complex computer language. All these factors combined facilitate the rapid development of bioinformatic tools that can perform quick and complex analyses of protein structures.

**Conclusion:**

p3d is the perfect tool to quickly develop tools for structural bioinformatics using the Python scripting language.

## Background

The increasing number of high-resolution protein structures available in the protein database [1] allows knowledge based approaches [2-4] by comparing structural features throughout non-redundant protein data sets [5]. Such knowledge based approaches can help to identify key parameters in e.g. ligand binding [6] or can be used to estimate favourable structural configurations that are important for de novo protein design or prediction of protein folding [7,8]. However, such approaches require a rapid development of new structural bioinformatic tools that are adapted to the questions asked, which in turn requires a robust framework or module. p3d is such a module for the Python scripting language . Although similar modules exist as part of the BioPython project [9] or part of the biskit package [10], p3d was developed in order to offer a Python module that is powerful and fast, yet intuitive to use. The simplicity of p3d is due to a) the usage of object oriented programming (i.e. atoms are treated as vectors), b) the implementation of a query parser that translates queries written in human readable language into a combination of algebra set operations and c) the fact that no additional Python packages are necessary. The speed is due to the usage of a binary space partitioning (BSP) tree which allows very fast queries in 3D [11]. The additional strength is obtained by the flexible combination of both speed and complexity in the intuitive and thus natural queries to the structural data.

The combination of these factors makes p3d the optimal module to rapidly develop new and powerful bioinformatic tools that follow the Python philosophy of making the source code readable.

## Implementation

**Figure 1:**
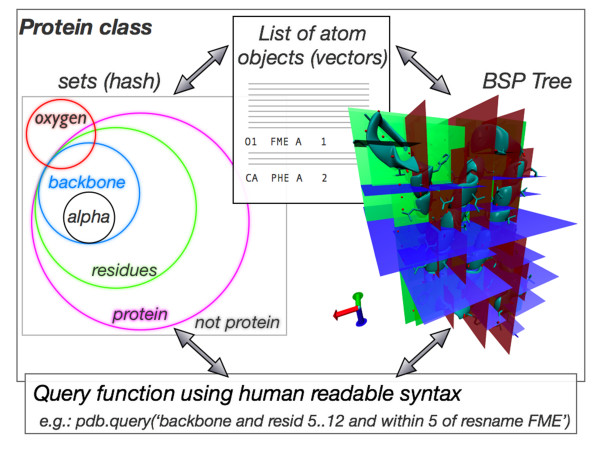
**p3d data structures**. The p3d data structures are a) a list of all atom objects that can be treated as vectors, b) a variety of sets, where each atom can be member of several sets and c) a Binary space-partitioning (BSP) tree that allows fast spatial queries to the protein structure. Illustrated are the recursive divisions performed on an aquaporin structure (Chain A, 1RC2.pdb [12]). Finally, the implemented query functions allow the combination of all three hashes and custom user defined vectors or atoms to formulate complex queries in a human readable syntax.

p3d is written in python 2.6 and compatible with the upcoming new standard Python 3.0. PDB files are read into the p3d structure, schematically illustrated in figure 1. During pdb loading each atom is converted into an atom object, which inherits all properties form the vector class (see below). These objects are stored into a list and linked to their proper sets, which will be used by the query function. Figure 1 shows some sets and how they intersect. Queries can therefore be directly translated into algebraic set operations, e.g. "select all atoms that are oxygens, belong to the residue name ATP and have a residue id smaller than 20". For fast queries in 3D a binary space partitioning (BSP) tree is generated automatically. There, the structure is divided into small subspaces. Figure 1 illustrates the recursive divisions performed on an aquaporin structure (Chain A, 1RC2.pdb [12]) during tree initialisation. The implemented query functions allow the combination of all sets, of the BSP tree neighbor search and of custom user defined vectors or atoms. Thus very complex queries can be formulated in a human readable syntax (see below).

## Results and Discussion

p3d offers an intuitive and robust interface between the Python scripting language and the complex nature of protein structure files. The input files can be in pdb format or the compressed gzip versions. All following examples, indicated with ">>>" are within the Python IDLE console, but can equally be incorporated into standalone scripts. A more detailed documentation for all modules and functions can be found online [13]. Loading a protein structure is done via:

>>> from p3d.protein import Protein

>>> pdb = Protein('2AXT.pdb.gz')

All atoms are treated as vector objects and can be rapidly accessed via hash tables, algebra of sets, lists, the BSP-tree class and any combination of those. For example all atoms are stored in the list *pdb.atoms *and the hash table can be found in the dictionary *pdb.hash*. Detailed information of how the structural data can be directly accessed can be found in the online documentation [13].

An easier and more intuitive access to the structural information is offered via the *query() *or *lookUpAtom() *functions. These functions try to return a set of atoms or one atom object, respectively. The usage for the query function is e.g.:

>>> atoms = pdb.query("chain A and resid 13..25")

This will return all atoms that are part of chain A and have a residue id from 13 to 25. The returned list of atom objects can then directly be used in another query (see below) or can be treated like vectors. The complete syntax of the query-string can be found in the online documentation [13].

Such a generalised approach brings a lot of flexibility and robustness. As a result, a lot of exceptions in pdb files can be handled without additional precautions, for example: a) Multiple models/structures (NMR, e.g. 7GAT[14]), b) Alternative side-chain conformers (e.g. 1BPH, [15]) or alternative main chain tracings (e.g. 1AZZ, [16]), c) disordered residues (e.g. 1EN2, (Saul et al. 2000)) and d) non amino acid residues, e.g. DNA (e.g. 7GAT[14]).

Benchmarking BioPython's pdb module against p3d showed that both modules have their strengths and weaknesses and, as usual, the results depend on the choice of the testing routines. For example, BioPython's pdb module performs a faster neighbour search since it calls a subroutine written in *c *from a different BioPython module whereas p3d relays on its BSP Tree that is written in Python and is as such slower. P3d is however faster in selecting wanted atoms due to its implementation of sets whereas BioPython's pdb module requires looping and unfolding over all entities. As a result p3d performs better if a neighbour search is connected with a complex query, such as all protein oxygen atoms within 3 Å of ATP simply because BioPython's pdb module requires a neighbour search for each ATP atom and additional checking if the found atom is part of the protein.

From a programming point of view, another clear advantage of p3d is its intuitive and simple usage, e.g. during benchmarking sample scripts written in p3d required one line using the query function while BioPythons pdb module required 7 lines including 4 loops over all pdb structure entities.

Another advantage of p3d is that each atom is treated as one object and no additional conversions or translations have to taken into account. The atom objects are created from each line in the pdb files. Each created Atom object holds all information regarding its properties, i.e. the Cartesian coordinates (x, y, z), the atom type, the residue name and number, the peptide chain and parent protein it is part of.

Therefore simple recursive queries through a protein structure can be performed, e.g.:

>>> atom = pdb.lookupAtom("resname ILE & oxygen & resid 30")

>>> chainAtoms = atom.allAtomsOfSameChain()

>>> residueAtoms = atom.allAtomsOfSameResidue()

Additionally the atom information of the alternative conformer labels, the model number (NMR structures) and the beta and user value are part of the atom class, e.g.:

>>> atom.x; atom.beta; atom.atype; atom.resid; atom.chain; atom.model

Since the Atom object inherits its properties form the Vector object, simple vector operations, such as addition, subtraction, length, dot and cross product are possible at the atom level without any additional overhead, e.g.:

>>> O = pdb.lookUpAtom("chain A & resid 1 & atype O")

>>> N = pdb.lookUpAtom("chain A & resid 2 & atype N")

>>> v = O + N; v = O - N; v = O.dot(N); v.length(); v = O.cross(N)

The history of vector operations on atoms is stored in the atom.desc property, thus allowing to keep a record on the performed transformations. Other implemented vector operations can be found in the online manual. The vector class can also be used to define new objects, new points of interest in space. Those can then be used as part of the query function. This interchangeability between structural data and user-defined vectors is unique to p3d. The user can therefore query protein surroundings by defined coordinates in a simple way, e.g.

>>> v = p3d.vector.Vector(18.00,12.00,-23.4)

>>> atomsAroundv = pdb.query("protein and within 4 of ", v)

The current version of p3d features two additional classes based on vector operations. These are the TransformationMatrix (TM) class and the Plane class. Both are part of the p3d.geo module. The TM class returns a matrix object when two sets of three vectors (source and destination) are given. Vectors or atoms that are multiplied with the matrix will be transformed from the source space into destination space. This can be used e.g. to align structures with only a few lines of source code:

>>> alignAtoms = ['N1','C5','N3']

>>> sourceAtoms = []

>>> targetAtoms = []

>>> for atom in alignAtoms:

>>> sourceAtoms.append(pdb1.lookUpAtom('resname ATP and atom type ', atom))

>>> targetAtoms.append(pdb2.lookUpAtom('resname ATP and atom type ', atom))

>>> tm = p3d.geo.TransformationMatrix(sourceAtoms, targetAtoms)

>>> for atom in pdb1.atoms:

>>> print (tm*atom).output()

The complete script can be found on the p3d web site [13].

The Plane class allows e.g. calculations of dihedral angles,

>>> p3d.geo.dihedral(atom1, atom2, atom3, atom4)

>>> alpha = pdb.lookUpAtom("alpha and resid 1 and chain A and model 2")

>>> alpha.calcPhiPsi()

Furthermore, the Plane class can for example also be used to calculate orientations of ligands over flat co-factors if three atoms of the co-factor are used to define the plane. This was used to calculated the orientation of histidine heme ligands relative to the heme by projecting a vector that represents the ligand orientation, (i.e. v = ND1 - CG) onto the heme plane, i.e. heme.projectionOfVector(ND1-CG) [7]. A basic example for this usage is:

>>> a = p3d.vector.Vector(1,0,0)

>>> b = p3d.vector.Vector(0,1,0)

>>> c = p3d.vector.Vector(1,1,0)

>>> plane = p3d.geo.Plane(a,b,c)

>>> k = p3d.vector.Vector(2,2,2)

>>> plane.projectionOfVector(k).info(lvl='coordinates')

[2.000, 2.000, 0.000]

All atom properties can be changed and the altered protein can be easily saved to a new file, e.g:

>>> for atom in pdb.atoms:

... atom.translateBy(k)

... atom.beta = 3.2

>>> pdb.writeToFile(pdb.fullname+'_changed.pdb')

The implementation of a BSP tree accelerates queries in space. The query functions allow the combination of spatial, i.e. BSP Tree queries and set theory, thus very complex queries can be formulated at ease, e.g.:

>>> ATPs = pdb.query("resname ATP")

>>> surrounding = pdb.query("protein & within 3 of ", ATPs, " and not nitrogens")

Example scripts shown online [13] illustrate furthermore the simplicity of Python scripts that use the p3d module. These are for example a script that analyses the distribution of phi and psi angles in a non-redundant protein set similar to the work of Hovmöller et al. [17]. By using p3d this analysis can be performed using only 26 lines of code. Another example is a script that determines the distances between different protein chains, which can be written with 36 lines of code, documentation included. This data can be used to plot the contact map between different protein chains.

Overall these features and their intuitive usage highlight the possibility to develop tools for structural bioinformatics rapidly.

## Future development

P3d will be kept updated and user requests might be implemented into the source code. Overall p3d will be maintained by the authors and hopefully other programmers will join this open source project. Two future aims will be a) to implement a faster BSPTree, eventually written in *c/c++ *and b) to add the syntax for spatial queries, e.g. select all "proteins and x-coordinates < 40". Furthermore p3d's website will expand with scripts that are posted by the users/readers.

## Conclusion

The p3d package extends the Python scripting language with a robust and powerful interface to investigate and manipulate protein structure files. The object oriented approach of p3d, the treatment of atoms as vectors, the usage of sets, the implementation of a BSP tree and the combination of all these factors into a query interface that uses human readable language make p3d a very fast and versatile module that allows rapid development of high throughput tools for structural bioinformatics.

## Availability and requirements

**Project name**: p3d

**Project home page**: 

**Operating systems(s)**: Platform independent

**Programming language**: Python 2.6+ and 3.0 ready

**Other requirements**: none

**License**: GNU GPL V2

**Any restrictions to use by non-academics**: none

## Authors' contributions

CF, concept, design, manuscript, coding of the protein, atom, vector, geo, protein, library and tree submodules and online manual. MS important contributions to the code design and coding of the query and geo module. All authors read and approved the final manuscript.
